# Expression of *phosphatase of regenerating liver* family genes during embryogenesis: an evolutionary developmental analysis among *Drosophila*, amphioxus, and zebrafish

**DOI:** 10.1186/1471-213X-13-18

**Published:** 2013-05-04

**Authors:** Ming-Der Lin, Hsun-Tzu Lee, Szu-Chieh Wang, Han-Ru Li, Hsin-Lun Hsien, Kai-Wen Cheng, Yu-Di Chang, Min-Lang Huang, Jr-Kai Yu, Yau-Hung Chen

**Affiliations:** 1 Department of Molecular Biology and Human Genetics, Tzu-Chi University, No.701, Zhongyang Rd., Sec 3, Hualien 97004, Taiwan; 2 Department of Life Sciences, Tzu-Chi University, No.701, Zhongyang Rd, Sec 3, Hualien 97004, Taiwan; 3 Department of Life Science, National Chung-Cheng University, No.168, Sec. 1, University Rd, Min-Hsiung Township, Chia-yi County 621, Taiwan; 4 Institute of Cellular and Organismic Biology, Academia Sinica, 128 Academia Road, Sec. 2, Nankang, Taipei 11529, Taiwan; 5 Department of Chemistry, Tamkang University, 151 Ying-Chuan Rd., Tamsui, New Taipei City 25137, Taiwan

**Keywords:** Phosphatase of regenerating liver, PTP4A, Embryogenesis, *Drosophila*, Zebrafish, Amphioxus

## Abstract

**Background:**

Phosphatase of regenerating liver (PRL) family is classified as class IVa of protein tyrosine phosphatase (PTP4A) that removes phosphate groups from phosphorylated tyrosine residues on proteins. PRL phosphatases have been implicated in a number of tumorigenesis and metastasis processes and are highly conserved. However, the understanding of PRL expression profiles during embryonic development is very limited.

**Results:**

In this study, we demonstrated and characterized the comprehensive expression pattern of *Drosophila PRL*, amphioxus *PRL*, and zebrafish *PRL*s during embryonic development by either whole mount immunostaining or *in situ* hybridization. Our results indicate that *Drosophila PRL* is mainly enriched in developing mid-guts and central nervous system (CNS) in embryogenesis. In amphioxus, initially *PRL* gene is expressed ubiquitously during early embryogenesis, but its expression become restricted to the anterior neural tube in the cerebral vesicle. In zebrafish, *PRL-1* and *PRL-2* share similar expression patterns, most of which are neuronal lineages. In contrast, the expression of zebrafish *PRL-3* is more specific and preferential in muscle.

**Conclusions:**

This study, for the first time, elucidated the embryonic expression pattern of *Drosophila*, amphioxus, and zebrafish *PRL* genes. The shared *PRL* expression pattern in the developing CNS among diverse animals suggests that *PRL* may play conserved roles in these animals for CNS development.

## Background

Phosphatase of regenerating liver (PRL) family is classified as class IVa of protein tyrosine phosphatase (PTP4A) that removes phosphate groups from phosphorylated tyrosine residues on proteins. Mammalian PRL family consists of three PRL members. PRL-1 (PTP4A1) was originally identified as an immediate-early growth response gene in the nucleus of regenerating rat liver and mitogen-treated 3T3 mouse fibroblasts [1,2]. PRL-2 (PTP4A2) and PRL-3 (PTP4A3) were subsequently discovered through database searches for sequences homologous to PRL-1 [3].

Overexpression of PRL family members has been implicated in cancer progression. For example, *PRL-3* up-regulation clearly correlates with colon carcinoma metastases, gastric carcinoma with nodal metastasis, ovarian carcinoma, breast carcinoma, and liver carcinoma cells (reviewed in [4]). Notably, *PRL-3* expression level in primary colorectal cancers has prognostic significance in predicting the development of liver and lung metastases [5]. *PRL-1* has been found to elevate in renal carcinoma, melanoma, pancreatic cancer cells, and ovarian lymphoma cells [6-8]. In addition to *PRL-1* and *PRL-3*, *PRL-2* overexpression is associated with prostate malignancies [9] and breast cancer [10,11]. These observations indicate that PRL family proteins play important roles in metastasis and a variety of cancers [4,12].

Although the PRL family members are known to be involved in cancer progression and metastasis, the understanding of normal PRL expression patterns during embryonic development is limited. Most of previous studies were focused on examining PRL expression in adult tissues. For example, rat *Prl-1* is mainly expressed in brain, skeletal muscle [1,13], and a number of digestive epithelial tissues [14,15]. Rat *Prl-2* mRNA is widely expressed in adult tissues including the anterior pituitary, brain cortex, adrenal gland, kidney, testis, and heart [16]. Rat PRL-3 protein has not yet been examined in adult normal tissues but is found to be expressed in fetal heart [17]. In mouse, *Prl-1* mRNA is expressed at all stages examined from E10.5 through E18.5 in a variety of tissues except heart and skeletal muscle [18]. In contrast, mouse *Prl-2* mRNA is preferentially expressed in skeletal muscle and *Prl-3* mRNA is mainly expressed both in the skeletal muscle and heart [3]. In addition, mouse PRL-3 is also expressed in *villus* epithelial cells of the small intestine [19]. Similar to mouse *Prl-1*, human *PRL-1* and *PRL-2* are almost ubiquitously expressed in adult human tissues, except that *PRL-1* is absent in the brain cortex [20]. In contrast to the ubiquitous expression pattern, human *PRL-3* mRNA is most enriched in the heart and skeletal muscle and moderately expressed in the pancreas [21]. These observations indicate that the expressions of PRL members can be varied in a tissue specific manner in mammals.

To our knowledge, no study has yet described and compared the expression patterns of PRLs in *Drosophila* and zebrafish model animals during embryonic development. Here, we demonstrated and characterized, for the first time, the comprehensive expression pattern of both *Drosophila PRL* and zebrafish *PRLs* during embryonic development by either whole mount immunostaining or *in situ* hybridization. To further understand the evolution of *PRL* gene in the chordate lineage, we also identify the single *PRL* orthologue in the basal chordate amphioxus and study its embryonic expression. Our study reveals evolutionary conserved as well as lineage specific *PRL* expression patterns during embryonic development among *Drosophila*, amphioxus, and lower vertebrate zebrafish.

## Results

### *Drosophila*, amphioxus, and zebrafish PRL phosphatases are highly conserved

PRL phosphatases from selected species and their molecular features are summarized in Table 1. All of these PRL phosphatases possess alkaline isoelectric points (pIs) and are proposed to be positively charged at physiological pH. In vertebrates, PRL family contains three PRL phosphatases whereas there is only one PRL gene in protostomes including *C. elegans* and *Drosophila*, and in invertebrate deuterostomes including sea urchin and amphioxus (Table 1)*.* Among vertebrates, PRL homologs including human, mouse, and zebrafish share more than 80% amino acid sequence identity. For instance, zebrafish PRL-1, PRL-2, and PRL-3 share 92%, 82%, and 88% identities with their human orthologs, respectively. Although a little bit lower, the protostome and invertebrate deuterostome PRLs also share significant amino acid sequence identity and similarity with their vertebrate orthologs. For instance, *Drosophila* PRL shares 75% similarity and 58% identity with human PRL-1 and 74% similarity and 57% identity with zebrafish PRL-1.

**Table 1:** Summary of PRL/PTP4A family proteins from selected species

**Gene names (species)**	**Coding region (aa)**	**Mw (kDa)**	**pI**	**GenBank accession number**
**Invertebrate PRL**				
*C. elegans*	190	21.16	8.97	NM_076282
*Drosophila*	176	19.97	8.83	NM_135936
Sea urchin	179	20.44	8.98	XM_001198915
(*S. purpuratus*)				
Amphioxus	174	19.9	9.07	KC_491215
(*B. floridae*)				
**Vertebrate PRL**				
**PRL-1**				
Human	173	19.82	9.17	NM_003463
Rat	173	19.82	9.17	NM_031579
Mouse	173	19.82	9.17	NM_011200
Zebrafish	173	20.00	9.33	NM_001007775
**PRL-2**				
Human	167	19.13	8.67	NM_080391
Rat	167	19.13	8.67	BC_060549
Mouse	167	19.12	8.68	NM_008974
Zebrafish	168	19.33	8.61	NM_001024098
**PRL-3**				
Human	173	19.53	9.35	NM_032611
Rat	173	19.65	9.42	NM_001114405
Mouse	173	19.65	9.42	NM_001166388
Zebrafish	173	19.88	9.45	NM_213181

**Figure 1:**
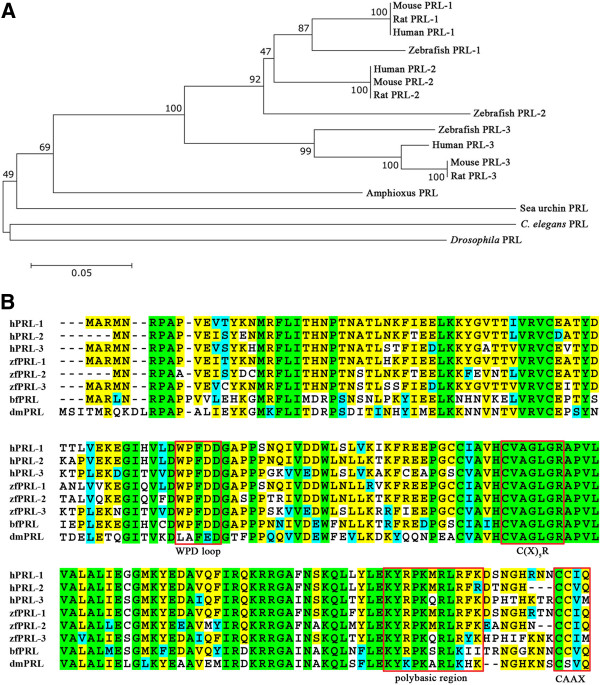
**Phylogenetic tree and multiple sequence alignment of PRL protein sequences from human, zebrafish, amphioxus, and *Drosophila*.** (**A**) Phylogenetic tree of PRL homologue proteins. PRL protein sequences from various species were retrieved from GenBank and corresponding accession numbers are provided in Table 1. The evolutionary history was inferred using the Neighbor-Joining method. The percentage of replicate trees in which the associated taxa clustered together in the bootstrap test (1000 replicates) are shown next to the branches. The tree is drawn to scale, with branch lengths in the same units as those of the evolutionary distances used to infer the phylogenetic tree. The evolutionary distances were computed using the p-distance method and are in the units of the number of amino acid differences per site. Evolutionary analyses were conducted in MEGA5. (**B**) Multiple sequence alignment of PRL protein sequences from human, zebrafish, amphioxus and *Drosophila.* Location of conserved catalytic site C(X)_5_R, WPD loop, polybasic region and prenylation motif CAAX box are indicated. Abbreviations: hPRL: *homo sapiens* PRL, zfPRL: zebrafish *Danio rerio* PRL, bfPRL: amphioxus *Branchiostoma floridae* PRL, dmPRL: *Drosophila melanogaster* PRL.

To get an idea about the evolutionary relationships of PRL phosphatases among species, we performed phylogenetic analysis. By using neighbor-joining (NJ) method [22] with p-distance model that conducted in MEGA5 [23], vertebrate PRL phosphatases were grouped as a robust clade with 100% bootstrap support (Figure 1A). This phylogenetic tree demonstrated that vertebrate *PRLs* originated through gene duplication. Besides, PRL-3 phosphatase is classified as a more distinct group from both PRL-1 and PRL-2 phosphatases. Interestingly, the basal chordate *Branchiostoma floridae* contains only one PRL phosphatase gene in its genome (Table 1), but we identified three cDNA isoforms from our cDNA/EST database. When we aligned these three PRL isoforms to the assembled draft genome sequence, we found they differ in their exon usage for the 5’ and 3’ UTR region, whereas the exon regions for the protein coding sequence are the same and thus encode an identical protein sequence consisting 174 amino acids (Additional file 1: Figure S1). The single *B. floridae* PRL sequence branches out at the base of all vertebrate PRLs, suggesting that the duplication events generating PRL-1, PRL-2, and PRL-3 occurred during early vertebrate evolution.

Previous studies indicate that PRL phosphatases contain conserved WPD loop and C(X)_5_R catalytic motif required for its phosphatase enzymatic activity [24,25]. In addition, the C-terminal region consists of a polybasic region together with a C-terminal prenylation motif which are required for its association with plasma membrane and early endosome [19,26,27]. The multiple sequence alignment reveals that *Drosophila*, amphioxus and zebrafish PRLs also contain these highly conserved signatures (Figure 1B).

### *Drosophila* PRL is expressed in developing mid-gut and CNS in embryogenesis and localized on the plasma membrane

**Figure 2:**
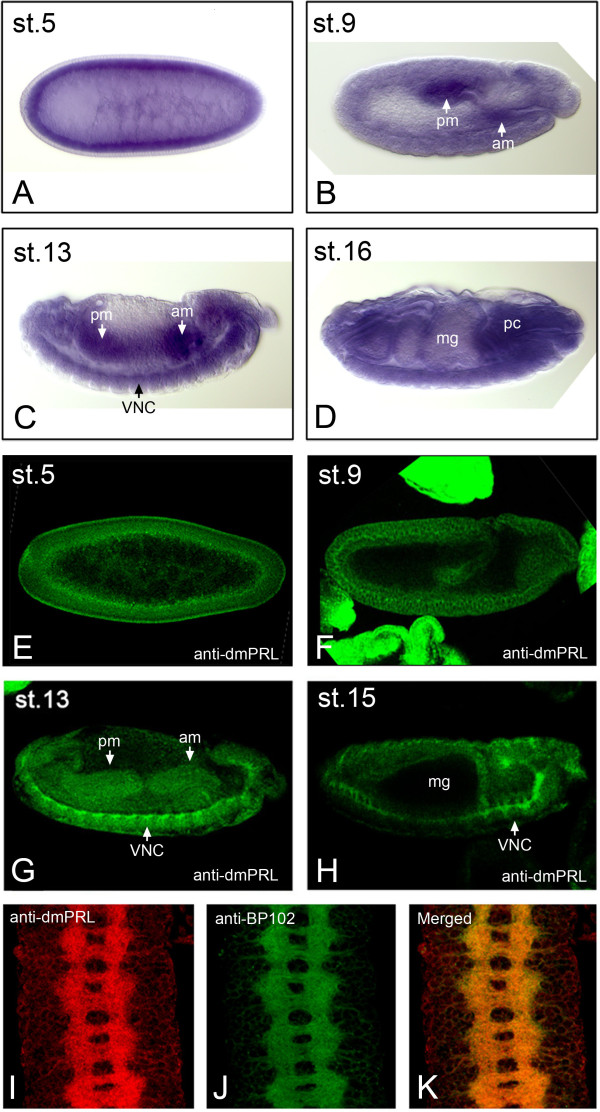
***Drosophila *PRL is enriched in developing mid-gut and the ventral nerve cord in embryogenesis.** (**A**-**D**) Whole mount *in situ* hybridization using antisense probes against *Drosophila PRL*. (**A**) *dmPRL* was ubiquitously expressed in stage 5 embryos. (**B**) At stage 9, *dmPRL* was expressed in developing anterior and posterior mid-guts. (**C**) At stage 13, *dmPRL* was expressed in developing anterior and posterior mid-guts together with VNC. (**D**) At stage 16, the expression of *dmPRL* was significant in procephalon. (**E-H**) Whole mount immunostaining of *Drosophila* PRL during embryogenesis. At stage 5 (**E**) and stage 9 (**F**), DmPRL was ubiquitously expressed. (**G**) At stage 13, dmPRL was expressed in developing anterior and posterior mid-guts together with VNC. (**H**) At stage 15, the expression of dmPRL was enriched in VNC. (**I-K**) dmPRL (**I**) and axon marker BP102 (**J**) were colocalized (**K**) in VNC of stage 15 embryos. Abbreviations: am, anterior mid-gut; pm, posterior mid-gut; VNC, ventral nerve cord; mg, mid-gut; pc, procephalon.

To analyze the expression pattern of *Drosophila PRL* (*dmPRL*) in embryogenesis, we performed whole mount *in situ* hybridization. At embryonic stage 5, *dmPRL* is ubiquitously expressed throughout the embryos (Figure 2A). In stage 9 embryos, *dmPRL* is expressed at developing anterior mid-gut and posterior mid-gut (Figure 2B, arrows). At stage 13, *dmPRL* expression is sustained in developing anterior mid-gut and posterior mid-gut (Figure 2C, arrows). In addition, the expression of *dmPRL* in ventral nerve cord (VNC) can also be observed at stage 13 embryos (Figure 2C, arrows). In stage 16 embryos, *dmPRL* was expressed in a significant amount in procephalon (Figure 2D).

To analyze the expression pattern and subcelluar localization of *Drosophila* PRL protein, we generated an anti-dmPRL antibody. The full length *dmPRL* CDS with 176 codons was subcloned into pET-32a vector for producing recombinant dmPRL proteins which were used to generate dmPRL antiserum from rabbits. The dmPRL antiserum preferentially recognized a protein band that represents dmPRL in embryo lysates around 22 kDa on Western blots (Additional file 2: Figure S2, arrowhead). The antibody specificity of dmPRL antiserum was further proved by its recognition of GFP-PRL fusion proteins in ovary lysate (Additional file 2: Figure S2, arrow). To explore the embryonic expression of dmPRL protein, we preformed whole mount immunostaining. At embryonic stage 5, dmPRL is mainly localized in the apical membrane of blastoderm embryos (Figure 2E). In stage 9 embryos, dmPRL is ubiquitously expressed in the germ band and evenly distributed in the plasma membrane of the cells (Figure 2F). At stage 13, the dmPRL expression is enriched in VNC (Figure 2G). In addition, dmPRL expression can be detected in the developing anterior and posterior mid-guts (Figure 2G, arrows). In stage 15 embryos, dmPRL expression persisted in the VNC (Figure 2H, arrow) but the mid-gut expression of dmPRL had vanished (Figure 2H). To confirm the expression of dmPRL in VNC, we dissected VNC of stage 15 embryos and performed double immunostaining with anti-dmPRL and anti-BP102, an axonal marker, antibodies. The dmPRL signal colocalized with BP102 staining perfectly (Figures 2I-K), indicating dmPRL expression is indeed in VNC. In summary, our data suggest that dmPRL begins to enrich in the axon of VNC by embryonic stage 13.

**Figure 3:**
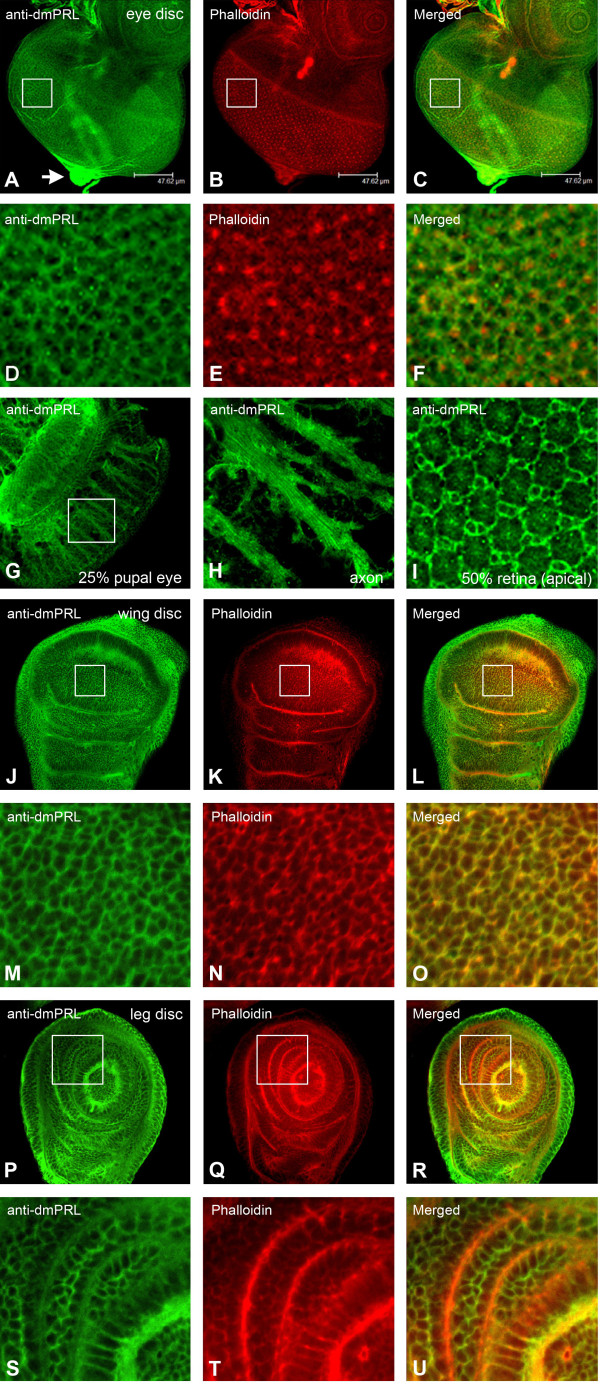
***Drosophila *PRL is localized on the plasma membrane of imaginal disc cells and enriched in developing axons of pupal retina.** (**A-F**) Expression of *Drosophila* PRL in larval eye imaginal discs. Eye antennal discs of 3^rd^ instar larvae were dissected and stained with anti-dmPRL antibody. DmPRL was expressed all over the eye discs but is more enriched in differentiating retinal cells (**A**). The arrow indicates the axon bundles of photoreceptor cells. In panel (**A-C**), the indicated insets are reproduced at (**D-F**) to show the enlarged view of the same field. DmPRL (**D**) was partially colocalized with phalloidin (**E**) in the differentiating eye disc cells (**F**). (**C** and **F**) Merged images. (**G-I**) DmPRL expression on developing pupal eye discs. In pupal discs at 25% pupal development, DmPRL was found to be enriched in developing axon (**G**). The indicated inset of (**G**) is reproduced at (**H**) to show the enlarged view of dmPRL staining. In pupal discs at 50% pupal development, dmPRL can be detected in the plasma membrane of developing retinal cells (**I**). (**J-O**) Expression of *Drosophila* PRL in larval wing imaginal discs. DmPRL was ubiquitously expressed throughout the wing discs (**J**). In panel (**M-O**), the indicated insets was reproduced at (**J-L**) to show the enlarged view of the same field. DmPRL (**J**) was colocalized with phalloidin (**K**). (**L** and **O**) Merged images. (**P-U**) Expression of *Drosophila* PRL in larval leg imaginal discs. DmPRL was ubiquitously expressed throughout the leg discs (**P**). In panel (**S-U**), the indicated insets was reproduced at (**P-R**) to show the enlarged view of the same field. DmPRL (**S**) was colocalized with phalloidin (**T**). (**R** and **U**) Merged images.

Previous study indicates that PRL phosphatases contain a C-terminal CAAX prenylation motif which is required for its association with plasma membrane [19]. To further confirm whether dmPRL is localized on the plasma membrane as its human homologs [19,28], we examined the expression of dmPRL on eye imaginal disc. In *Drosophila* eye disc, dmPRL is more prominent in differentiating disc cells (Figures 3A and D) and colocalized with phalloidin (Figures 3B and E) that marks filamentous actin. This result indicates that dmPRL is associated with plasma membrane as its human homologs. In addition, we noticed an enrichment of dmPRL signal in the optic stalk which consisting of photoreceptor axons (Figure 3A, arrow). As the larval eye disc will develop into the future retina, we next examined developing pupal retinas. Similar to larval eye disc, dmPRL signal is highly enriched in the developing axons of pupal eye disc at 25% pupal development (Figures 3G-H). Further, we also observed that dmPRL can be clearly detected in the plasma membrane of pupal retina at 50% pupal development (Figure 3I). To examine whether imaginal discs other than eye disc can express dmPRL, we stained wing and leg imaginal discs. In wing discs, dmPRL was ubiquitously expressed (Figures 3J and M) and colocalized with phalloidin (Figures 3K and N). In leg discs, we also observed the ubiquitous expression of dmPRL (Figures 3P and S) and its colocalized with phalloidin (Figures 3Q and T). In conclusion, dmPRL is a membrane associated protein and its expression is enriched in developing mid-guts and VNC in embryogenesis.

### Zebrafish *PRL-1* and *PRL-2* are mainly expressed in neuronal cell lineages

**Figure 4:**
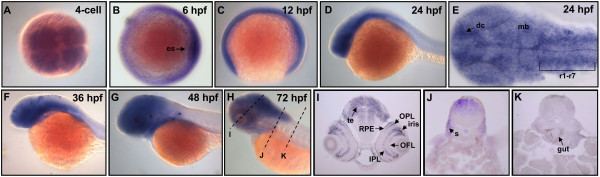
**Expression pattern of zebrafish *PRL-1 *during embryonic development.** (**A-D**, and **F-H**) Whole mount *in situ* hybridization using probes against zebrafish *PRL-1*. Dorsal view of 4-cell stage (**A**) and 6-hpf (**B**) embryos. Lateral view of 12-hpf (**C**), 24-hpf (**D**), 36-hpf (**F**), 48-hpf (**G**), and 72-hpf (**H**) embryos. (**E**) Flat mount showed zebrafish *PRL-1* signals in the entire brain. (**I-K**) Cross-sections along the plane indicated by lines showed in (**H**). Abbreviations: hpf, hours post fertilization; OPL, outer plexiform layer; OFL, optic fiber layer; IPL, inner plexiform layer; RPE, retinal pigment epithelium; dc, diencephalon; mb, midbrain; te, tectum; s, somite.

To determine the spatiotemporal expression patterns of *PRL-1, PRL-2* and *PRL-3* during early development of zebrafish, we performed whole mount *in situ* hybridization using antisense DIG-labeled riboprobes. Zebrafish *PRL-1* transcripts were first detected from 1~4-cell stages to cleavage period (Figure 4A), and extended their expression from the gastrula period to the early segmentation stages (Figures 4B-C). At 24-hpf, the zebrafish *PRL-1* transcripts were restricted to the head region (Figure 4D). Flat-mount of 24-hpf embryos revealed that *PRL-1* signals distributed in the entire brain, including the diencephalon, midbrain, and rhombomeres 1-7 (Figure 4E). By 36- (Figure 4F), 48- (Figure 4G) and 72-hpf (Figure 4H), zebrafish *PRL-1* expressed strongly in brain, eyes as well as in pharyngeal arches (Figures 4F-H). Cryosections of 72-hpf embryo revealed zebrafish *PRL-1* signals were detected at tectum, iris, retinal pigment epithelium, outer plexiform layer, inner plexiform layer, optic fiber layer (Figure 4I). However, the expression zebrafish *PRL-1* was relatively weak in somite (Figure 4J) and gut (Figure 4K). These results suggest that the expressions of zebrafish *PRL-1* transcripts are most abundant at neuronal lineages.

**Figure 5:**
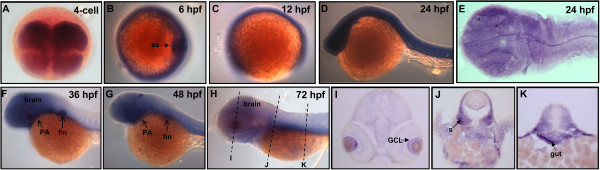
**Expression pattern of zebrafish *PRL-2 *during embryonic development.** (**A-D**, and **F-H**) Whole mount *in situ* hybridization using probes against zebrafish *PRL-2*. Dorsal view of 4-cell stage (**A**) and 6-hpf (**B**) embryos. Lateral view of 12-hpf (**C**), 24-hpf (**D**), 36-hpf (**F**), 48-hpf (**G**), and 72-hpf (**H**) embryos. (**E**) Flat mount showed zebrafish *PRL-2* signals in the entire brain. (**I-K**) Cross-sections along the plane indicated by lines showed in (**H**). Abbreviations: hpf, hours post fertilization; GCL, ganglion cell layer; s, somite.

Zebrafish *PRL-2* transcripts were first observed from 1~4-cell stages to cleavage period, and their expression extended from the gastrula period to the early segmentation stages (Figures 5A-C). By 24-hpf, the zebrafish *PRL-2* transcripts were detected in head region as well as in trunk (Figures 5D-E). By 36- (Figure 5F), 48- (Figure 5G) and 72-hpf (Figure 5H), zebrafish *PRL-2* expressed strongly in brain, eyes, fin buds as well as in pharyngeal arches (Figures 5F-H). Cryosections of 72-hpf embryo revealed *PRL-2* signals at outer layer of lens (Figure 5I, arrow), anterior somites (Figure 5J, arrow), and gut (Figure 5K, arrow), but were undetectable at retinal pigment epithelium, inner plexiform layer, optic fiber layer (Figure 5I). On the basis of these observations, we conclude that the neuronal expressions of zebrafish PRL-2 are similar to that of zebrafish *PRL-1*, except that zebrafish *PRL-1* displays stronger signals in eye regions. In contrast to zebrafish *PRL-1*, zebrafish *PRL-2* is strongly expressed in gut.

### Zebrafish *PRL-3* transcripts are expressed in brain, somites, blood island, and head muscles in developing embryos

**Figure 6:**
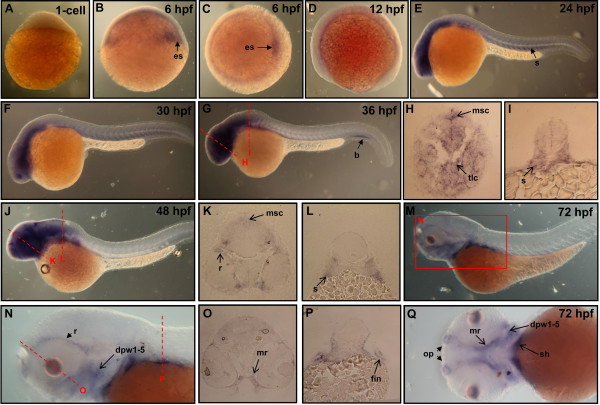
**Expression pattern of zebrafish *PRL-3 *during embryonic development.** (**A-Q**) Whole mount *in situ* hybridization using probes against zebrafish *PRL-3*. Dorsal view of a 6-hpf (**C**) embryo. Lateral view of 1-cell (**A**), 6-hpf (**B**), 12-hpf (**D**), 24-hpf (**E**), 30 hpf (**F**), 36-hpf (**G**), 48-hpf (**J**), and 72-hpf (**M**, **N**) embryos. (**H-I, K-L** and **O-P**) Cross-sections along the plane indicated by lines showed in (**G, J**, and **N**), respectively. (**Q**) Ventral view of a 72-hpf embryo. Abbreviations: b, blood island; dpw1-5, dorsal pharyngeal wall 1-5; es, embryonic shield; mr, medial rectus; msc, mesencephalon; s, somite; sh, sternohyoideus; pa, pharyngeal arch; r, retina; op, olfactory placode; tlc, telencephalon.

Zebrafish *PRL-3* transcripts were first observed at 1-cell stage (Figure 6A). At gastrula stage, *PRL-3* signals distributed in germ ring and in embryonic shield (es) (Figures 6B-C). By 12-hpf, the zebrafish *PRL-3* transcripts were down-regulated suggesting that maternal inherited *PRL-3* might be exhausted (Figure 6D). At 24-hpf (Figure 6E), 30-hpf (Figure 6F), and 36-hpf (Figure 6G), the zebrafish *PRL-3* transcripts were detected at head region. Cryosections of 36-hpf embryo reveals the expression of zebrafish *PRL-3* transcripts in brain region of mesencephalon and telencephalon (Figure 6H, arrows) and somites (Figure 6I, arrow). Afterward, *PRL-3* signals at head region persisted while its expression in somite diminished (Figures 6G-H). It is interesting to note that zebrafish *PRL-3* was transiently expressed at blood island (b) at 36 hpf (Figure 6G, arrow), suggesting that *PRL-3* might play a role on blood cell maturation. By 72-hpf, zebrafish *PRL-3* signals were expressed at a small subset of head muscles including dorsal pharyngeal wall 1-5 (Figure 6Q, arrow), medial rectus (Figures 6O and Q, arrows), sternohyoideus (Figure 6Q, arrow). In addition, zebrafish *PRL-3* signals can be observed in retina (Figure 6N, arrow), fin (Figure 6P, arrow), and olfactory placode (Figure 6Q, arrow).

### Amphioxus *PRL* is initially expressed throughout the embryo but later concentrated to the anterior neural tube in the larva

**Figure 7:**
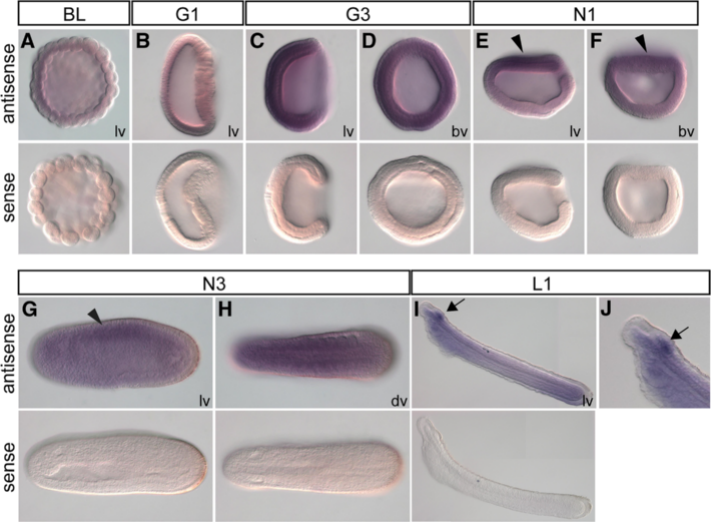
**Expression pattern of amphioxus *PRL *during embryonic development.** (**A-I**) Upper images show results of whole mount *in situ* hybridization using antisense probes against amphioxus *PRL*; lower images are whole mount *in situ* hybridization using sense amphioxus *PRL* probes as negative controls. (**A**) Blastula stage, (**B**) early gastrula stage, (**C-D**) mid gastrula stage, (**E-F**) early neurula stage, (**G-H**) late neurula stage, (**I**) 36-hpf larval stage; lv, lateral view; bv, blastopore view; dv, dorsal view; arrowheads indicate weak expression in the dorsal part of the embryos; arrow in (**I**) indicates expression in the cerebral vesicle. (**J**) Enlarged view of the anterior end of the same 36-hpf larva in (**I**) showing amphioxus *PRL* expression in the cerebral vesicle (arrowhead).

To understand the evolution of *PRL* developmental expression patterns, we analyzed *PRL* gene expression in basal chordate amphioxus by whole mount *in situ* hybridization. At blastula stage, weak amphioxus *PRL* expression was detected throughout the entire embryo (Figure 7A), and this ubiquitous expression continued to gastrula stage (Figure 7B-D). At neurula stages, amphioxus *PRL* transcripts were still detected throughout the embryo, but we noticed that the signals appeared to be stronger in the dorsal side of the embryos (Figure 7E-H, arrowheads). During the early larval stage, we found amphioxus *PRL* transcripts were more concentrated in the anterior neural tube at the cerebral vesicle of amphioxus (Figure 7I-J), although some weak signals were still detectable throughout the body.

## Discussion

PRL is an evolutionary conserved protein family that is widely distributed in many species among the protostomes and deuterostomes. Through database search, it is evident that all of the examined vertebrate genomes possess three *PRL* genes, whereas invertebrate animals including *C. elegans*, *Drosophila*, Sea urchin, and the basal chordate amphioxus *Branchiostoma floridae* have only one *PRL* gene. Our molecular phylogenetic analysis suggests that the three vertebrate *PRL* genes may be the product of gene duplication events that happened at the base of the vertebrate lineage. By comparing whole-genome synteny patterns, it has been shown that the vertebrate genome had undergone two rounds of whole-genome duplication during early vertebrate evolution [29]. We analyzed the synteny patterns between amphioxus and human genomic regions around *PRL* genes and found that the single amphioxus *PRL* gene is located on the *Branchiostoma floridae* genomic scaffold 233 (Additional file 3: Figure S3A), while the three human *PRL* paralogues are located on three different chromosomes. We identified some conserved synteny patterns among amphioxus *PRL* and human *PRL-1* and *PRL-2* (Additional file 3: Figure S3B); however, no synteny conservation could be identified around the genomic region harboring *PRL-3* on human chromosome 8, which may be due to genomic rearrangement after duplication. In fact, we also detected extensive genomic rearrangements that may have caused translocation (such as *MDGA1* on human chromosome 6) and tandem duplication of genes (such as *AGO1*, *AGO3* on *AGO4* on human chromosome 1) around the vertebrate *PRL-1* and *PRL-2* genomic regions (Additional file 3: Figure S3B), suggesting that after two rounds of whole-genome duplication, vertebrate genomes have experienced extensive rearrangements during evolution.

In mammals, some of the expression profiles of *PRL* phosphatase have been characterized at either the protein and/or mRNA levels in normal tissues (Table 2). However, embryonic expression patterns of PRL orthologs have not yet been characterized in teleosts and invertebrates. In this study, we characterized all of the three zebrafish *PRL* genes, *Drosophilia PRL*, as well as amphioxus *PRL*, and examined their embryonic expression patterns. In vertebrates including zebrafish, rat, mouse, and human, most of PRL-1 and PRL-2 are found to be expressed in the neuronal cell lineage (Table 2 and Figures 4 and 5). With regard to *Drosophila* PRL, it was detected strongly in the ventral nerve cord during embryogenesis (Figure 2), and the expression of amphioxus PRL gene is also detected in the anterior central nervous system (Figure 7). These results suggest that the biological role of “prototypic PRL” might be involved in the nervous system development.

**Table 2:** **Expression profiles of *PRL *family genes in embryos, larvae, and adult normal tissues**

**Species**	**Gene**	**Embryonic and/or larval expression domains**	**Expression in adult tissue**
**Rat**	*PRL-1*	Brain [13]	In brain, skeletal muscle, and digestive epithelial tissues [1,13-15]
	*PRL-2*	ND	Ubiquitous expression [16]
	*PRL-3*	Fetal heart [17]	ND
**Mouse**	*PRL-1*	Most tissues except heart and skeletal muscle [18]	ND
	*PRL-2*	ND	Preferential in skeletal muscle [3]
	*PRL-3*	ND	Preferential in heart and skeletal muscle; also in small intestine [3,21]
**Human**	*PRL-1*	ND	Ubiquitous expression except brain cortex [19]
	*PRL-2*	ND	Ubiquitous expression [19]
	*PRL-3*	ND	Mainly in heart and skeletal muscle; pre-erythrocytes in bone marrow [17,20]
**Zebrafish**	*PRL-1*	Mainly in neuronal cell lineage	ND
	*PRL-2*	Mainly in neuronal cell lineage	ND
	*PRL-3*	Mainly in skeletal muscle	ND
**Amphioxus**	*PRL*	Ubiquitous in embryos; Central nervous system in larvae	ND
***Drosophila***	*PRL*	Developing mid-gut and central nervous system in embryos; Imaginal discs in larvae	ND

Abbreviation: ND, Not determined.

In contrast to general neuronal expression of *PRL-1*, *PRL-2*, the expression of mammalian and zebrafish *PRL-3* phosphatases are preferential in mesodermal cell lineage, such as heart, skeletal muscle, and pre-erythrocytes (Table 2 and Figure 6). Thus, we propose that the ancestral *PRL* is predominantly expressed in neuronal cell lineage, especially in the central nervous system. In the vertebrate lineage, two of the duplicated *PRL* paralogues, *PRL-1* and *PRL-2*, retained this neuronal expression, while the third paralogue, *PRL-3*, may have evolved new function and obtained more specific expression in the mesodermal cell lineage.

## Conclusions

In summary, our study characterizes the distinct expression patterns of three structurally related zebrafish *PRL* genes and those of *Drosophila* and amphioxus *PRL* homologues. Our comparisons provide interesting insight into the evolution of *PRL* genes and their embryonic expression patterns, and highlight the possible consequence of gene duplication events on the neofunctionalization of duplicated genes during vertebrate evolution.

## Methods

### Antibody generation

To generate a rabbit anti-PRL antibody, we subcloned the full length dmPRL CDS from cDNA clone RE40268 into pET-32a vector for producing recombinant His-tagged dmPRL proteins in *E.coli*. The dmPRL recombinant protein purification together with the generation of the anti-dmPRL polyclonal rabbit antiserum were performed by GeneTex International Corp. approved by the Industrial Development & Investment Promotion Committee of Hsin-Chu City, Taiwan.

### *Drosophila* whole mount immunostaining

For embryo immunostaining, collected embryos were washed with 0.4% NaCl, 0.1% Triton X-100, dechorionated with 100% bleach for 2 min, and washed with deionized water. Embryos were fixed for 20 min in 4% formaldehyde with heptane. After removal of the fixative, embryos were washed with methanol several times and rehydrated into phosphate buffered Tris. Embryos were blocked with 2% bovine serum albumin in PBT (PBS containing 0.2% Tween-20) for 1 hour, and incubated overnight at 4°C in primary antibody diluted in PBS. The embryos were then washed 3 times for 20 min each in PBT, and then incubated for 2 h at room temperature in secondary antibody in PBT. Following three 30 min washes in PBT, the embryos were mounted in anti-fade mounting solution (PBS containing 50% glycerol and 2% DABCO). For eye disc immunostaining, hand dissected eye discs were fixed for 20 min in 4% formaldehyde. After removal of the fixative, embryos were washed several times in PBST (PBS containing 0.3% Triton X-100). Eye discs were blocked with 2% bovine serum albumin in PBST for 1 hour, and incubated overnight at 4°C in primary antibody diluted in PBS. The eye discs were then washed 3 times for 20 min each in PBST, and then incubated for 2 h at room temperature in secondary antibody in PBST. Following three 30 min washes in PBT, the discs were mounted in anti-fade mounting solution. The following primary antibodies were used: rabbit anti-PRL, rat anti-ELAV (Developmental Studies Hybridoma Bank), mouse anti-HRP (Jackson Labs). Fluorescent-labeled secondary antibodies used goat-anti-rabbit Alexa Fluor 488 (Invitrogen), goat anti-rat Alexa Fluor 633 (Invitrogen), and goat anti-mouse Alexa Fluor 633 (Invitrogen). F-actin was labeled by Alexa Flour 633 conjugated phalloidin (Invitrogen).

### *Drosophila* whole mount *in situ* hybridization

The full length CDS of *dmPRL* cDNA were cloned into pGEM-3Z vector and used to synthesize digoxigenin (DIG) labeled RNA probes for *in situ* hybridization. Template preparation, probe synthesis, and procedure for whole mount *in situ* hybridization were performed as previously described [30]. All embryos were observed under a Nikon Eclipse E800 microscope equipped with Nomarski differential interference contrast optics and a CCD camera.

### Zebrafish strain and embryo staging

Mature zebrafish (AB strain) were raised at the zebrafish facility of the Life Sciences Development Center, Tamkang University. All animal experiments in this study were approved by Tamkang University and performed in accordance with the “Animal Research: Reporting in vivo Experiments” guideline issued by regional animal ethic committee. The fish were maintained at 28°C with a photoperiod of 14 h light and 10 h dark, in an aquarium supplied with freshwater and aeration [31]. Embryos were produced using standard procedures and were staged according to standard criteria [32].

### Zebrafish whole mount *in situ* hybridization, cryosection and imaging

The procedures for whole mount *in situ* hybridization, and cryosection have been described previously [33], except that the full length of individual zebrafish *PRL-1, PRL-2,* and *PRL-3* coding sequences were used to produce digoxigenin-labeled probes. All embryos were observed under a Leica DM 2500 microscope equipped with Nomarski differential interference contrast optics (Kramer Scientific) and a digital camera (Cannon, Japan).

### Identification of amphioxus *PRL* homologue and whole mount *in situ* hybridization

Amphioxus homologue of *PRL* gene was identified from *Branchiostoma floridae* draft genome [29] by BLAST search using *Drosophila* PRL protein as queries. Identified Gene models were subsequently used to search the amphioxus EST database and cDNA collection (*B. floridae* Gene Collection Release 1 [34]) to isolate the corresponding cDNA clones. The identified cDNA clones were sequenced from both ends by M13 forward and reverse primers as well as internal primers to obtain the complete nucleotide sequence of the inserts. At the end, we identified three different cDNA isoforms (amphioxus cDNA ID: bfad016c08, bfad039b03, and bfad043g05) representing one single *PRL* gene in amphioxus (see Results).

Gravid animals of the amphioxus (*B. floridae*) were collected in Tampa Bay, Florida USA, during the summer breeding season. Gametes were obtained by electric stimulation. Fertilization and subsequent culturing of the embryos were carried out as previously described [35]. Amphioxus *PRL* cDNA clones isolated from the aforementioned cDNA collection were used to synthesize digoxigenin (DIG) labeled anti-sense and sense RNA probes for *in situ* hybridization. Template preparation, probe synthesis, and procedure for single-color *in situ* hybridization were performed as previously described [36]. Whole-mount *in situ* hybridization on amphioxus embryos was performed as previously described [37]. Images of embryos were taken using a Zeiss Axio Imager A1 microscope with a Zeiss AxioCam MRc CCD camera. Probes generated from the three amphioxus *PRL* cDNA isoforms gave us identical pattern, thus we only present results from one cDNA clone, bfad016c08, which gave us the strongest signals.

## Abbreviations

CDS: Coding sequence; CNS: Central nervous system; DIG: Digoxigenin; hpf: Hours post fertilization; PRL: Phosphatase of regenerating liver; VNC: Ventral nerve cord.

## Competing interests

The authors declare that they have no competing interests.

## Authors’ contributions

MDL, JKY, and YHC conceived and designed the experiments. HTL, SCW, HRL, HLH, KWC, YDC, and MLH performed the experiments. MDL, JKY, and YHC wrote the paper. All authors read and approved the final manuscript.

## Supplementary Material

Additional file 1: Figure S1 Genomic structure of amphioxus *PRL* gene. The thick black line represents the 33kb genomic DNA region on scaffold Bf_V2-223 that contains the *PRL* locus. Grey boxes above the genomic scaffold are the locations of the predicted exons. Patterns of exon usage for each cDNA isoforms are depicted with colored boxes under the genomic scaffold. Red lines indicate the position of start and stop codon. Scale bar: 1kb. Click here for file

Additional file 2: Figure S2 Anti-dmPRL rabbit antiserum recognized both endogenous dmPRL and GFP-tagged dmPRL in ovary and embryo lysates. (**A**) Anti-dmPRL rabbit antiserum recognized both the GFP-tagged dmPRL fusion protein (lane 1, arrow) and endogenous dmPRL (lane 1-3, arrowheads) in both ovary and embryo lysates. (**B**) The same PVDF membrane of (**A**) was stripped and re-probed with anti-GFP antibody to show the GFP-dmPRL fusion protein of GFP-dmPRL expressing ovary lysate. (**C**) The same PVDF membrane of (**A**) was stripped and re-probed with anti-Tubulin antibody as a loading control. Asterisk indicates the non-specific band recognized by anti-dmPRL rabbit antiserum. Lane 1: GFP-dmPRL expressing ovary lysate. Lane 2: wild-type ovary lysate. Lane 3: wild-type embryo lysate. Click here for file

Additional file 3: Figure S3 Synteny comparison between amphioxus and human *PRL* chromosomal regions. (**A**) The single amphioxus *PRL* gene is located on Scaffold 233 in Version 2 assembly (scaffold_104 of Version 1 assembly in the JGI genome browser). Arrows represent the amphioxus *PRL* and its neighboring genes on the scaffold in the direction of transcription. The numbers represent the distance (in kilo-base) between neighboring genes. In this schematic drawing the distances between genes are not in scale. (**B**) The three human *PRL* paralogues (*PRL-1*, *PRL-2*, and *PRL-3*) are located on three different chromosones. Black arrows highlight the positions of *PRL* paralogues and the traces of conserved synteny between amphioxus and human *PRL* chromosome region. Human synteny information was retrieved from NCBI Map Viewer database at http://www.ncbi.nlm.nih.gov/mapview/ Click here for file
