# Supplementary material for: Expression of phosphatase of regenerating liver family genes during embryogenesis: an evolutionary developmental analysis among Drosophila, amphioxus, and zebrafish
Source: BMC Dev Biol. 2013 May 4;13:18. doi: 10.1186/1471-213X-13-18 (PMC3663695; doi:10.1186/1471-213X-13-18)
Supplement: Additional file 1: Figure S1 — Genomic structure of amphioxus PRL gene. The thick black line represents the 33kb genomic DNA region on scaffold Bf_V2-223 that contains the PRL locus. Grey boxes above the genomic scaffold are the locations of the predicted exons. Patterns of exon usage for each cDNA isoforms are depicted with colored boxes under the genomic scaffold. Red lines indicate the position of start and stop codon. Scale bar: 1kb. [file 1471-213X-13-18-S1.pdf]

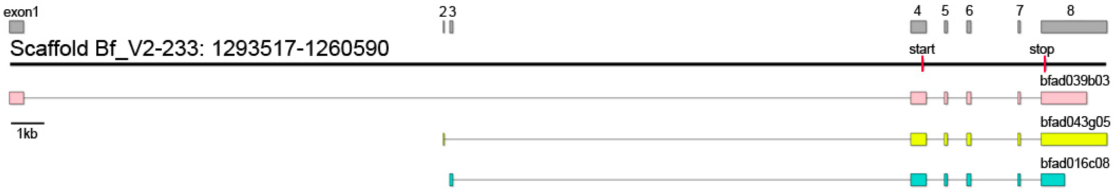

Figure S1. Genomic structure of amphioxus *PRL* gene.

The thick black line represents the 33kb genomic DNA region on scaffold Bf_V2-223 that contains the *PRL* locus. Grey boxes above the genomic scaffold are the locations of the predicted exons. Patterns of exon usage for each cDNA isoforms are depicted with colored boxes under the genomic scaffold. Red lines indicate the position of start and stop codon. Scale bar: 1kb.
