# Supplementary material for: Expression of phosphatase of regenerating liver family genes during embryogenesis: an evolutionary developmental analysis among Drosophila, amphioxus, and zebrafish
Source: BMC Dev Biol. 2013 May 4;13:18. doi: 10.1186/1471-213X-13-18 (PMC3663695; doi:10.1186/1471-213X-13-18)
Supplement: Additional file 2: Figure S2 — Anti-dmPRL rabbit antiserum recognized both endogenous dmPRL and GFP-tagged dmPRL in ovary and embryo lysates. (A) Anti-dmPRL rabbit antiserum recognized both the GFP-tagged dmPRL fusion protein (lane 1, arrow) and endogenous dmPRL (lane 1-3, arrowheads) in both ovary and embryo lysates. (B) The same PVDF membrane of (A) was stripped and re-probed with anti-GFP antibody to show the GFP-dmPRL fusion protein of GFP-dmPRL expressing ovary lysate. (C) The same PVDF membrane of (A) was stripped and re-probed with anti-Tubulin antibody as a loading control. Asterisk indicates the non-specific band recognized by anti-dmPRL rabbit antiserum. Lane 1: GFP-dmPRL expressing ovary lysate. Lane 2: wild-type ovary lysate. Lane 3: wild-type embryo lysate. [file 1471-213X-13-18-S2.pdf]

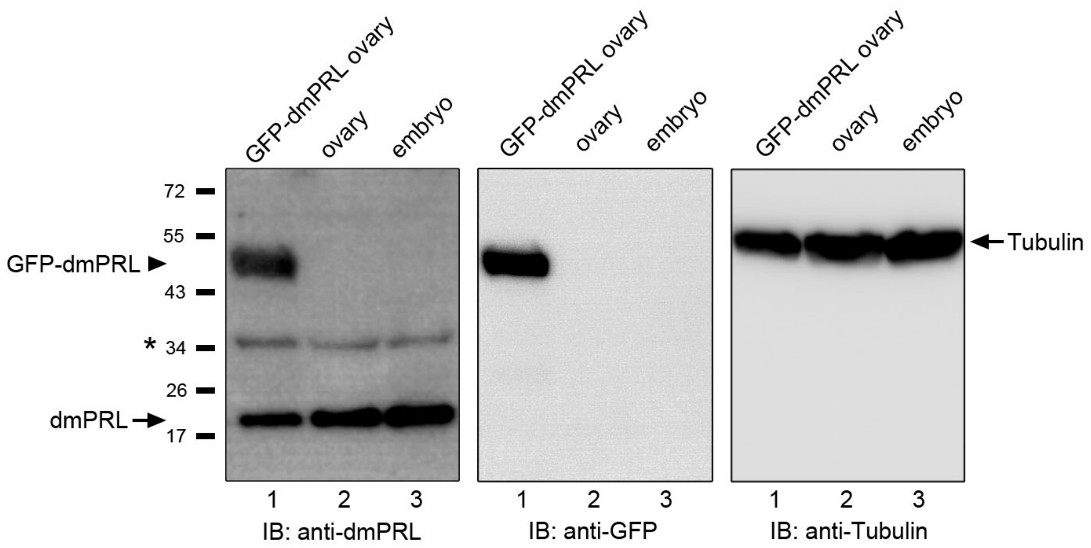

Figure S2. Anti-dmPRL rabbit antiserum recognized both endogenous dmPRL and GFP-tagged dmPRL in ovary and embryo lysates.

(A) Anti-dmPRL rabbit antiserum recognized both the GFP-tagged dmPRL fusion protein (lane 1, arrow) and endogenous dmPRL (lane 1-3, arrowheads) in both ovary and embryo lysates. (B) The same PVDF membrane of (A) was stripped and re-probed with anti-GFP antibody to show the GFP-dmPRL fusion protein of GFP-dmPRL expressing ovary lysate. (C) The same PVDF membrane of (A) was stripped and re-probed with anti-Tubulin antibody as a loading control. Asterisk indicates the non-specific band recognized by anti-dmPRL rabbit antiserum. Lane 1: GFP-dmPRL expressing ovary lysate. Lane 2: wild-type ovary lysate. Lane 3: wild-type embryo lysate.
