# Supplementary material for: Expression of phosphatase of regenerating liver family genes during embryogenesis: an evolutionary developmental analysis among Drosophila, amphioxus, and zebrafish
Source: BMC Dev Biol. 2013 May 4;13:18. doi: 10.1186/1471-213X-13-18 (PMC3663695; doi:10.1186/1471-213X-13-18)
Supplement: Additional file 3: Figure S3 — Synteny comparison between amphioxus and human PRL chromosomal regions. (A) The single amphioxus PRL gene is located on Scaffold 233 in Version 2 assembly (scaffold_104 of Version 1 assembly in the JGI genome browser). Arrows represent the amphioxus PRL and its neighboring genes on the scaffold in the direction of transcription. The numbers represent the distance (in kilo-base) between neighboring genes. In this schematic drawing the distances between genes are not in scale. (B) The three human PRL paralogues (PRL-1, PRL-2, and PRL-3) are located on three different chromosones. Black arrows highlight the positions of PRL paralogues and the traces of conserved synteny between amphioxus and human PRL chromosome region. Human synteny information was retrieved from NCBI Map Viewer database at http://www.ncbi.nlm.nih.gov/mapview/ [file 1471-213X-13-18-S3.pdf]

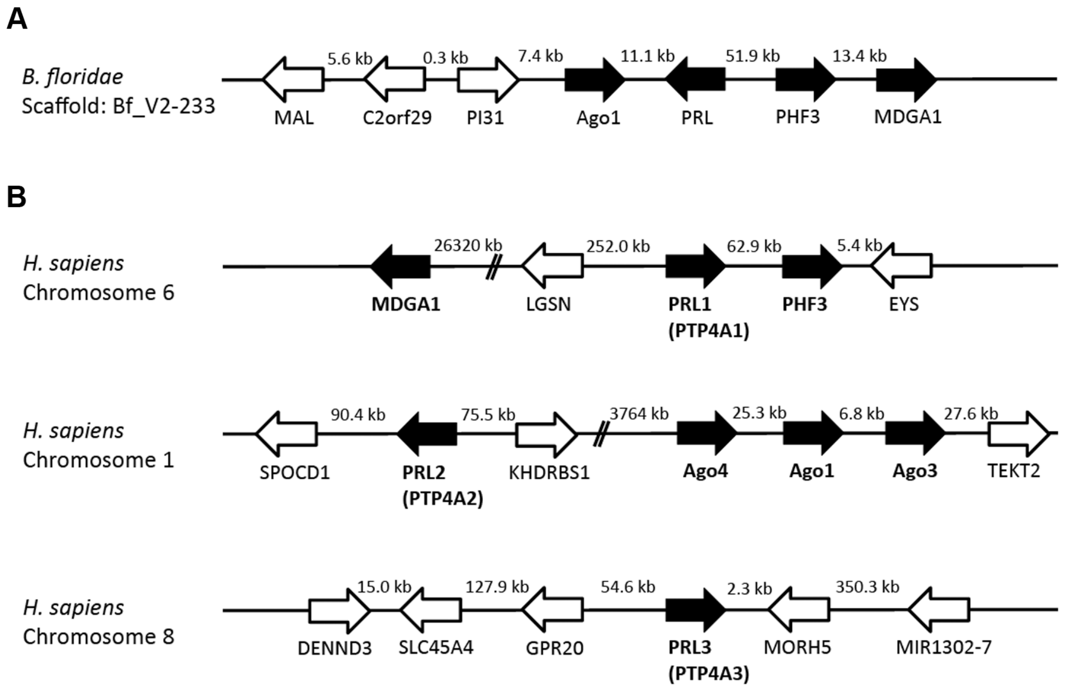

Figure S3. Synteny comparison between amphioxus and human *PRL* chromosomal regions.

(A) The single amphioxus *PRL* gene is located on Scaffold 233 in Version 2 assembly (scaffold_104 of Version 1 assembly in the JGI genome browser). Arrows represent the amphioxus *PRL* and its neighboring genes on the scaffold in the direction of transcription. The numbers represent the distance (in kilo-base) between neighboring genes. In this schematic drawing the distances between genes are not in scale. (B) The three human *PRL* paralogues (*PRL-1*, *PRL-2*, and *PRL-3*) are located on three different chromosones. Black arrows highlight the positions of *PRL* paralogues and the traces of conserved synteny between amphioxus and human *PRL* chromosome region. Human synteny information was retrieved from NCBI Map Viewer database at http://www.ncbi.nlm.nih.gov/mapview/.
